# Efficacy and Safety of Long-acting Injectable Cabotegravir and Rilpivirine in Improving HIV-1 Control in sub-Saharan Africa: Protocol for a Phase 3b Open-Label Randomized Controlled Trial (IMPALA)

**DOI:** 10.12688/wellcomeopenres.23363.1

**Published:** 2025-03-28

**Authors:** Victoria Babirye Tumusiime, Eugene Ruzagira, Claire Norcross, Ingrid Eshun-Wilsonova, Jonathan Kitonsa, Ubaldo Mushabe Bahemuka, Daniel Grint, Geofrey Kimbugwe, Ayoub Kakande, David S Lawrence, Fridah Mwendia, Rodica Van Solingen, Veerle Van Eygen, Fafa Addo Boateng, Fiona Cresswell, IMPALA study team, Idahosa Awonukhe, Noami Waita, Willemijn Rein VanDerHorst, Derrick Addy, Emilija Tomic, Noela Clara Owarwo, Eva Laker, Sylivia Cornelia Nassiwa, Ibrahim Yawe, Adolf Alinaitwe, Henry Mugerwa, Loice Achieng Ombajo, Joseph Nkuranga, Nigel Garrett, Sharana Mahomed, Bongi Zuma, Nirosha Gokul, Sheetal Kassim, Dorothie VanderVendt, Fatim Abrahams, Ruanne Barnabas, Jessie Heitner, Agnes Ssali, Dominic Bukenya, Violet Ankunda, Ayub Kakande, Charles Ogwang, Priscilla Balungi, Jane Nabbuto, Florence Nambaziira, Charliene Katurole, Jascent Nassuna, Diana Gibb, Cissy Kityo, Chloe Orkin, Simiso M Sokhela, Ivy Kaunda, Geofrey Kimbugwe, Paddy Kafeero, Phiona Nabaggala, Mina Ssali Nakawuka, Joseph Lutaakome, Gloria Lubega, Moses Kibirige, Gertrude Nabulime, Margaret Nalaaki, Peace Bukirwa, Deus Wangi, Dorothy Abigaba, Antonio Ssewakambo, Daniel Opoka, Slyvia Namaganda, Henry Rogers Isiko, Max Okwero, Annet Nankya, Diana Namuddu, Hamza Mayanja, Patricia Nerima, Jesca Asienzo, Doerieyah Reynolds, Agatha Theuri, Elizabeth Kamau, Felix Humwa, Jacqueline Nyange, Simon Wahome, Eve Koile, Susan Wanjiru Maina, Susan Onywera, Victor Omodi, Edwin Othieno, Arnold Onyango, Gerald Kiambi, Martha Atandi, Beryl Handa, Amos Ingubo, Fahkri Williams, Yashna Singh, Melanie Maclachlan, Zareen Dhansay, Sherisce Govender, Sumaiyya Moosa, Viwe Soko, Awethu Fuzie, Rochel Jacobs, Mary Sihlangu, Yamkela Sapepa, Monica Vogt, Upasna Singh, Nicola Bodley, Lorna Dias, Carol Minnie, Mpume Khoza, Renita David, Lungile Maziya, Silindile Molefe, Nokuphiwa Zondi, Amanda Khuzwayo, Nivashnne Naicker, Atika Moosa, Nomfundo Mbatha

**Affiliations:** 1 HIV interventions, MRC/UVRI and LSHTM Uganda Research Unit, Entebbe, Central Region, 256, Uganda; 2 Department of Infectious Diseases Epidemiology, London School of Hygiene and Tropical Medicine Faculty of Epidemiology and Population Health, London, England, UK; 3 Global Health and Infectious, Brighton and Sussex Medical School, Brighton, England, UK; 4 Johnson & Johnson, CapeTown, South Africa; 5 Department of Clinical Research, London School of Hygiene and Tropical Medicine Faculty of Infectious and Tropical Diseases, London, England, UK; 6 Botswana Harvard Health Partnership, Gaborone, Botswana; 7 School of Pathology, Faculty of Health Sciences , University of Witwatersrand, Johannesburg, South Africa; 8 Johnson & Johnson, East Africa, Nairobi, Kenya; 9 Jansenn Research and Development, Beerse, Belgium; 10 Johnson & Johnson Middle East FZ LLC, Accra, Ghana

**Keywords:** Treatment adherence, Long-acting, Cabotegravir, Rilpivirine, HIV-1, Antiretroviral therapy

## Abstract

Long-acting (LA) injectable therapy as treatment for HIV-1 infection offers reduced dosing frequency, increased discretion and provides an alternative to two or three-drug daily oral combinations. A parenteral LA formulation of cabotegravir (CAB) and rilpivirine (RPV) given by intramuscular (IM) injection every 2 months (CAB LA + RPV LA Q2M) has shown safety and efficacy in phase 3/3b trials and could increase treatment satisfaction and improve adherence in sub-Saharan Africa.

IMPALA (IMProving HIV-1 Control in Africa using Long-Acting Antiretrovirals) is a randomized, controlled, open-label, multicentre, interventional study in Uganda, Kenya, and South Africa of 540 virologically suppressed adults living with HIV-1 infection with a history of sub-optimal adherence to daily oral ART . IMPALA seeks to demonstrate the non-inferior antiviral effectiveness of switching to CAB LA + RPV LA Q2M, compared to the continuation of first-line daily oral ART containing 2 nucleoside/nucleotide reverse transcriptase inhibitors (NRTIs) plus dolutegravir (DTG). After providing informed consent, participants are screened for eligibility. Those who are viraemic (HIV RNA ≥200 copies/mL) at screening will be suppressed (<200 copies/mL for ≥3 months) on oral ART prior to randomization. On Day 1, individuals will be randomized 1:1 to either continue daily oral ART, or switch to CAB LA + RPV LA Q2M, (intervention arm) with or without a 1-month oral lead consisting of once daily oral CAB + RPV. The total follow-up period is 24 months. The primary endpoint is HIV-1 RNA <50 copies/ml at 12 months by FDA snapshot.

Initial: TMC278LAHTX3005 Version Amendment 1, 30 September 2022

Superseded by: TMC278LAHTX3005 Version Amendment 2, 3 June 2024

This clinical trial was registered on Clinical Trials.gov on 19 September 2022 (NCT05546242), available from https://clinicaltrials.gov/ct2/show/NCT05546242.

The study was also registered on Pan African Clinical Trials Registry on 23 January 2023 (PACT202301600757432TMC278LAHTX3005), available from https://pactr.samrc.ac.za/TrialDisplay.aspx?TrialID=24291.

## Plain language summary

The IMPALA study is an open-label phase 3b randomized clinical trial in Uganda, Kenya, and South Africa. It includes 540 adults with HIV-1 who have previously struggled to take daily HIV pills and have had high levels of HIV virus in their blood. Study participants have the virus suppressed with HIV pills and are then assigned, by chance, to either continue their daily oral HIV pills or switch to the long-acting HIV treatment comprising two drugs – cabotegravir and rilpivirine – which are given as two separate intramuscular injections every two months. Participants are followed for 24 months, and the trial will measure whether the injectable treatment is just as effective and safe as the daily oral treatment. The main goal is seeing if the virus remains suppressed after 12 months and comparing these results between the oral and injectable treatment.

## Introduction

### Background & study rationale

The purpose of the IMPALA study is to compare the effectiveness of long-acting (LA) intramuscular (IM) injectable cabotegravir (CAB) (ViiV) plus LA rilpivirine (RPV) (Johnson & Johnson) (CAB LA + RPV LA) versus continuation of daily oral standard of care (SOC) antiretroviral therapy (ART) in African adults with a history of sub-optimal HIV-1 control.

Despite documented increases in ART coverage over the last decade, advanced HIV disease/AIDS (CD4 <200 cells/μL) and opportunistic infections are still a major problem in sub-Saharan Africa. The majority of AIDS-defining conditions now occur in ART-experienced individuals with treatment failure, largely due to challenges with compliance with taking the daily oral pill required for effective HIV treatment ^1, 2^. This population remains viraemic in the community, risking onwards HIV transmission and perpetuating the HIV epidemic. Many individuals finally present to health services *in extremis* with opportunistic illnesses, which require protracted hospitalization resulting in high cost of care and contributing to the significant burden of AIDS-related mortality ^3, 4^.

In the UNAIDS 95-95-95 targets, the 3 ^rd^ ‘95’ relates to 95% of people on antiretroviral therapy (ART) achieving virologic suppression by 2030 ^5^. Although this figure stood at 93% in 2023, it translated to 72% of all people living with HIV (PLWH) globally ^4^. Further simplifying treatment and overcoming the need for daily pill taking is an important step in increasing treatment compliance, viral suppression, improving quality of life and reducing the death and disability caused by HIV treatment failure ^6^.

Long-acting (LA) injectable ART offers a reduced dosing frequency and the potential to improve HIV control and avert morbidity and mortality in those with sub-optimal adherence to daily oral ART ^7^. The combination regimen of CAB LA + RPV LA has been developed for maintenance of viral suppression in PLWH and has proven to be non-inferior to continuation of daily oral ART in Phase 3/3b clinical trials conducted predominantly in the global north ^8– 10^.

Recent clinical trial data from Sub-Saharan Africa confirm its non-inferior efficacy in settings with reduced frequency of HIV viral load (VL) monitoring and reduced availability of baseline HIV drug resistance testing, in individuals with a history of continued viral suppression ^11^. As well as demonstrating non-inferior viral control up to 152-weeks of follow-up, prior trial data suggest the treatment has high satisfaction rates, with over 95% of trial participants preferring CAB LA + RPV LA to daily oral therapy ^12, 13^. The study participants generally found the CAB LA + RPV LA injections to be acceptable and tolerable. Although injection site reaction (ISRs) were common, serious and severe ISRs were infrequent ^12, 13^. CAB LA + RPV LA is approved and marketed by ViiV as CABENUVA ^®^ (co-packaged product) in the US, Canada and Australia, and as 2 separate drugs: REKAMBYS ^®^ and VOCABRIA ^®^, in the EU and several other countries worldwide.

Evidence from public health approaches to HIV care in sub-Saharan Africa is needed to inform policy. The public health approach to HIV care and treatment provides a limited selection of ART regimens and monitoring services at no cost to the user at the point of access. A study modelling the potential impact of CAB LA + RPV LA roll-out in sub-Saharan African settings suggested that use of the regimen would reduce AIDS related mortality. The IMPALA study will build upon data being generated in the ‘Cabotegravir and Rilpivirine Efficacy and Safety’ (CARES) study which is ongoing in Uganda, Kenya, and South Africa ^14^. CARES is a Phase 3b randomized, open label trial in virologically suppressed (<50 copies/mL) HIV-1 positive adults, designed to demonstrate the non-inferior antiviral effect of switching to CAB LA + RPV LA every 2 months (Q2M) compared to continuation of first-line oral ART. Recently published 48 week outcome data from the CARES study have demonstrated CAB LA + RPV LA Q2M to be non-inferior to daily oral ART in this population ^11^. Adults who have been viraemic (HIV-1 VL ≥200 copies/mL) whilst on ART were excluded from CARES. The IMPALA study specifically recruited adults who have had a HIV VL >1000 copies/mL in the prior 2 years despite being on first-line oral ART, suggesting a history of adherence challenges, but being virologically suppressed for ≥3 months prior to randomization visit and without a baseline HIV drug resistance test. IMPALA intends to bridge this data gap on whether CAB LA + RPV LA can be used in individuals with a history of sub-optimal adherence and across different viral sub-types in sub-Saharan Africa.

### Hypotheses

The primary hypothesis is that CAB LA + RPV LA Q2M is non-inferior to SOC daily oral ART (2 NRTI + DTG) in maintaining viral suppression in people with a history of sub-optimal HIV-1 control in sub-Saharan Africa.

The secondary hypotheses are that CAB LA + RPV LA Q2M is safe, and well tolerated when compared to daily oral ART.

## Protocol

### Study design

**Figure 1.**
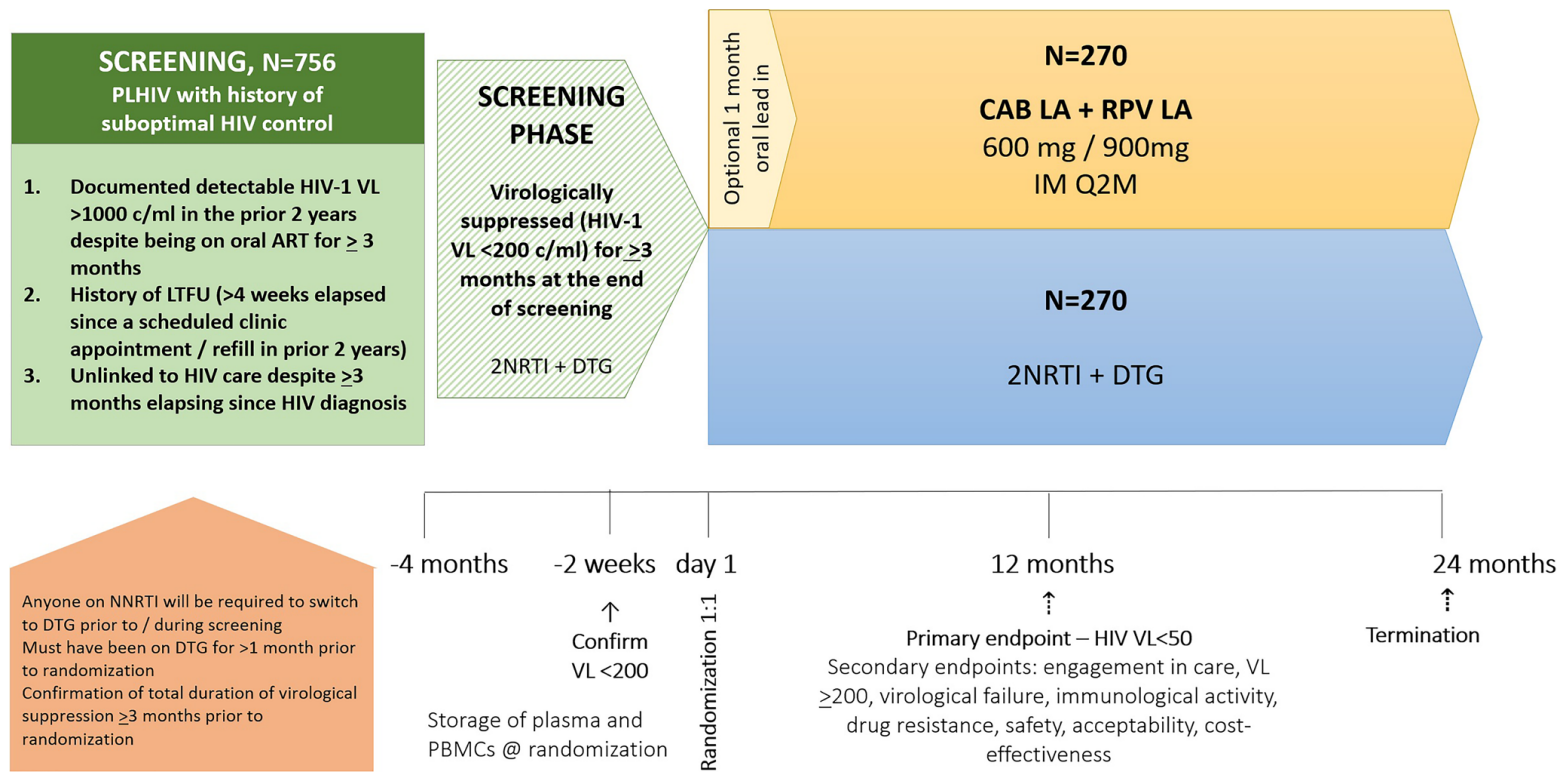
Schematic overview of the IMPALA Trial.

IMPALA is a randomized controlled open-label interventional study among virologically suppressed (HIV-1 viral load (VL) <200 copies/mL) adults (≥18 years) who have a history of sub-optimal adherence to daily oral ART and/or engagement in HIV care. The study aims to demonstrate non-inferior antiviral efficacy of switching to CAB LA + RPV LA Q2M in comparison to continuation of first-line daily oral ART containing 2 NRTIs + DTG. A schematic overview of the study design is shown in Figure 1.

The study target is 540 participants (270 participants per intervention group). Participants were recruited across sites from three countries (Uganda, Kenya and South Africa). The list of study sites is available from the trial registration on clinicaltrials.org. The trial population comprises participants with no significant laboratory abnormalities at screening, negative hepatitis B status, willing to adhere to suitable methods of contraception and with no known history of non-nucleoside reverse transcriptase inhibitor (NNRTI) and/or integrase strand transfer inhibitor (INSTI) resistance. The detailed list of inclusion and exclusion criteria is shown in Table 1.

**Table 1.** IMPALA Trial Inclusion and Exclusion Criteria.

IMPALA Trial Inclusion and Exclusion Criteria	
Inclusion Criteria	
**1.**	18 years of age and above.
**2.**	HIV-1 infection confirmed in clinic records or by study team.
**3.**	Virologically suppressed (HIV VL <200 copies/mL) for ≥3 months prior to randomization visit
**4.**	Is on a first-line oral regimen of 2NRTI + DTG
**5.**	Is identified as a participant with a history of, or at risk of, sub-optimal ART adherence or engagement in care based on one or more of the following criteria: a. Documented detectable HIV-1 viral load (>1000 c/mL) on all-oral ART (EFV/NVP or DTG-based) in the prior 2 years despite being ART-experienced for >3 months. b. History of being lost to follow-up from care (>4 weeks elapsed since a missed scheduled clinic appointment or refill in the prior 2 years). c. Failed to link to HIV care despite ≥3 months elapsed since HIV diagnosis
**6.**	Females: human chorionic gonadotrophin (HCG) negative and willing to use one highly effective form of contraception if WORP
**7.**	Male participants are advised to wear a condom for sexual intercourse.
**8.**	Must sign informed consent form (ICF) indicating that the participant understands the purpose of, and procedures required for, the study and is willing to participate in the study.
**9.**	Willing and able to attend all clinic appointments.
Exclusion Criteria	
**1.**	Not virologically suppressed (VL <200 c/mL) for >3 months at the conclusion of screening process.
**2.**	Previous use, or intention to use, protease inhibitor-based ART at any time.
**3.**	Evidence of prior HIV-1 resistance test with NNRTI drug resistance mutations (other than K103N) and/or INSTI drug resistance mutations.
**4.**	Unwillingness to receive two injections on a 2 monthly basis.
**5.**	Unwilling to use a form of contraception.
**6.**	Pregnant, breastfeeding or planning to become pregnant during the study period.
**7.**	Requires tuberculosis therapy or other drug with clinically relevant drug interaction (for a detailed listing of permitted and prohibited concurrent medications, refer to Section 6.8).
**8.**	High risk of seizures, including participants with an unstable or poorly controlled seizure disorder.
**9.**	Has active TB or other mycobacterial disease and requires treatment.
**10.**	Advanced liver disease, known biliary abnormalities (with the exception of Gilbert’s syndrome / gallstones) or history of cirrhosis.
**11.**	Chronic Hepatitis C with planned or anticipated use of Hep C therapy.
**12.**	Evidence of hepatitis B virus (HBV) infection based on the results of testing at Screening for Hepatitis B surface antigen (HBsAg), Hepatitis B core antibody (HBcAb), Hepatitis B surface antibody (HBsAb) and HBV DNA as follows: a. Participants positive for HBsAg are excluded b. Participants negative for HBsAb but positive for HBcAb, and negative for HBsAg, regardless of HBV DNA are excluded (NOTE: Participants positive for HBcAb (negative HBsAg status) and positive for HBsAb (past and/or current evidence) are immune to HBV and are not excluded)
**13.**	Current or anticipated need for chronic anti-coagulation therapy.
**14.**	Previous use of oral or injectable CAB or RPV.
**15.**	Any grade 4 laboratory abnormality at the conclusion of screening process.
**16.**	Creatinine clearance (CrCl) <50 mL/min/1.732 by Chronic Kidney Disease Epidemiology Collaboration (CKD EPI) equation
**17.**	Alanine transaminase (ALT) > 3× upper limit of normal (ULN)
**18.**	Has a tattoo or other dermatological condition overlying the gluteus region that may interfere with interpretation of ISRs
**19.**	Has ongoing or clinically significant medical conditions that in the opinion of the investigator may interfere with the absorption, distribution, metabolism or excretion of the study interventions or could affect participant safety.
**20.**	Has preexisting physical or mental condition which, in the opinion of the investigator, may interfere with the participant’s ability to comply with the dosing schedule and/or protocol evaluations or which may compromise the safety of the participant.
**21.**	Known allergies, hypersensitivity, or intolerance to cabotegravir or rilpivirine or its excipients.
**22.**	Received an investigational intervention (including investigational vaccines) or used an invasive investigational medical device within 30 days before the planned first dose of study intervention or is currently enrolled in another interventional study
**23.**	Has received any prohibited medication as specified in the protocol
**24.**	Has been treated with any of the following agents within 28 days of Screening: a. Radiation therapy b. Cytotoxic chemotherapeutic agents d. Tuberculosis therapy with the exception of isoniazid (isonicotinyl hydrazid, INH) e. Anticoagulation agents (e.g., warfarin and direct oral anticoagulants).
**25.**	Immunomodulators that alter immune responses (such as chronic systemic corticosteroids, interleukins, or interferons). (Note: Participants using short-term steroid tapers, topical, inhaled and intranasal corticosteroids, topical imiquimod are eligible)
**26.**	Treatment with an HIV-1 immunotherapeutic vaccine within 90 days of Screening
**27.**	QTc interval >450ms (if QTc interval is >450ms on the ECG read out, then it should be corrected according to Fridericia; https://www.mdcalc.com/corrected-qt-interval-qtc) within 90 days prior to study entry

Initial: TMC278LAHTX3005 Version Amendment 1, 30 September 2022

Superseded by: TMC278LAHTX3005 Version Amendment 2, 3 June 2024

This clinical trial was registered on Clinical Trials.gov on 19 September 2022 (NCT05546242), available from https://clinicaltrials.gov/ct2/show/NCT05546242.

The study was also registered on Pan African Clinical Trials Registry on 23 January 2023 (PACT202301600757432TMC278LAHTX3005), available from https://pactr.samrc.ac.za/TrialDisplay.aspx?TrialID=24291.

### Interventions

Screening for eligible participants is performed, and the baseline visit (Day 1) will occur within 4 months of the first screening visit. After participant information is provided an assessment of understanding is performed. Those who understand the implications of the study and are keen to participate are invited to provide written informed consent and are evaluated for eligibility during the screening period. Participants who are viraemic (HIV-1 VL ≥200 copies/mL) at the time of screening must be virologically suppressed on a regimen of 2 NRTIs + DTG for a minimum of 3 months prior to randomization.

Eligible participants are randomly assigned to one of 2 treatment arms using a computer-generated randomization schedule prepared in advance by the trial statistician. To ensure balance, randomization employs randomly permuted blocks of random block sizes (4, 6, 8, 10, 12) and stratified according to the study site. Based on this randomization code, the study investigational product is packaged and labelled.

On Day 1, virologically suppressed (<200 copies/mL for at least 3 months) individuals are randomized 1:1 to either continue daily oral ART (2 NRTI + DTG, control arm), or switch to Q2M CAB LA + RPV LA IM, the intervention arm. Those randomized to the intervention arm are offered either an optional oral lead in (OLI) of 1 month daily oral CAB and RPV or a direct to injection (DTI) approach. This decision to dose with or without an OLI phase is determined by the study participant following the informed consent discussion with the investigator. Baseline assessments are performed on Day 1.

### Intervention arm (CAB LA + RPV LA)

The participants who opt for the OLI Phase receive the study intervention in 2 phases:

*Oral Lead-in (OLI) Phase:* Starting on Day 1, participants receive CAB 30 mg plus RPV 25 mg once daily for 4 weeks to be taken at approximately the same time each day with a meal. The purpose of the OLI Phase is to determine individual tolerability of the combination prior to administration of CAB LA + RPV LA. Both OLI and DTI options have comparable safety and efficacy profiles ^15^.

*Maintenance Phase:* After the 4-week OLI Phase, participants return for the Month 1 visit to receive the first IM CAB LA 600 mg + RPV LA 900 mg initiation injections. The second initiation injections with CAB LA 600 mg + RPV LA 900 mg are administered at Month 2, and then continuation injections are administered every 2 months (Q2M) thereafter.

Participants who opt for direct to injection (DTI) (i.e. without the OLI phase) remain on daily oral ART for 4 weeks following randomization. They receive their first initiation injections of CAB LA + RPV LA at the Month 1 visit. The second initiation injections of CAB LA + RPV LA are administered at Month 2, followed by continuation injections CAB LA + RPV LA every 2 months thereafter.

The participants receive CAB LA + RPV LA via two separate 3 ml injections, one administered into each gluteal muscle, using a ventrogluteal approach. The ventrogluteal approach is recommended as the gluteus medius muscle is located away from major nerves and blood vessels ^16, 17^, and is more likely to avoid inadvertent subcutaneous administration. Injections are administered by appropriately trained staff and participants must be observed for 10–15 minutes post-injection to monitor for any immediate adverse reactions.

Under exceptional circumstances, to address pre-planned missed CAB LA + RPV LA dosing visits, the use of oral therapy as a “bridging” strategy is permitted. This would usually be in the form of daily oral CAB (30mg) + RPV (25mg), but alternative bridging therapy with local SOC can be considered, for instance when obtaining oral CAB and oral RPV is not practical.

### Comparator Arm (Oral cART)

Participants take a regimen of 2 NRTIs + DTG as per local country guidelines up to Month 24. If toxicity occurs, participants may switch daily oral ART drugs, but only after prior discussion with the Sponsor.

### Permitted Medications and Non-drug Therapies

Chemoprophylaxis for HIV-associated conditions is permitted, where required, based on the participant’s and their physician’s judgement. All concomitant medications, blood products, and vaccines administered during the study period will be documented in the eCRF, alongside administration dates. It is recommended that PLHIV are vaccinated against HBV, vaccination status should be discussed, and vaccination is encouraged in trial participants if not immune.

Since non-HIV vaccines can cause a temporary rise in plasma HIV-1 RNA levels, it is recommended that any vaccine deemed necessary (including approved COVID vaccines) be administered either during or right after a scheduled visit, once all laboratory test samples have been collected. This approach will reduce the risk of non-specific increases in plasma HIV-1 RNA at the next scheduled assessment.

Other intramuscular injectables (IM), with some exceptions below, are allowed but must be given at least 2 cm away from the site of the IP IM injection.

CAB oral administration: Products containing divalent cations (such as aluminium, calcium, and magnesium) should be taken at least 2 hours before or at least 4 hours after CAB.

RPV oral administration: Antacid products must be taken at least 2 hours before or at least 4 hours after RPV. H2-Receptor antagonists (e.g., cimetidine, famotidine, nizatidine, ranitidine) may cause significant decreases in RPV plasma concentrations. H2-receptor antagonists should only be administered at least 12 hours before or at least 4 hours after RPV. RPV should not be co-administered with proton pump inhibitors, such as esomeprazole, lansoprazole, omeprazole, pantoprazole, rabeprazole.

Administration of clarithromycin, erythromycin, and telithromycin is not recommended with RPV due to possible increase in plasma concentration of RPV due to CYP3A enzyme inhibition. Where possible, alternatives such as azithromycin should be considered.

Concurrent administration of multivitamins is acceptable.

### Prohibited Medications and Non-drug Therapies

The following concomitant medications or therapies are not permitted at any time during the study:

•      HIV immunotherapeutic vaccines are not permitted at any time during the study.

•      Other experimental agents, antiretroviral drugs not otherwise specified in the protocol, cytotoxic chemotherapy, or radiation therapy may not be administered.

•      Systemically administered immunomodulators (such as interleukin and interferon agents) are prohibited. This includes topical agents that have substantial systemic exposure and effects. The short-term use (30 days or less) of topical imiquimod is allowed.

•      Acetaminophen (paracetamol) is not permitted for participants with acute viral hepatitis.

•      Long-term use of systemic (oral or parenteral) glucocorticoids is not permitted due to their immunosuppressive effect and potential reduction in RPV plasma levels. However, short courses of oral prednisone, prednisolone, methylprednisolone (e.g., ≤14 days) are permitted. A single dose of systemic dexamethasone is allowed (multiple doses may significantly reduce RPV plasma concentration and are not permitted) and topical, inhaled or intranasal glucocorticoids are permitted.

•      Hepatitis C infection therapy is prohibited during the Maintenance Phase before the Month 12 primary endpoint, and interferon-based hepatitis C virus (HCV) therapy or use of any drugs that have a potential for adverse DDIs with study intervention is prohibited throughout the entire study.

For information on concurrent therapies and interactions suspected to be relevant to daily oral ART, please consult the Summary of Product Characteristics (SmPC) for the specific drugs.

### Prohibited Medications with CAB and/or RPV

For participants receiving either formulation of CAB and/or RPV, the following medications could significantly decrease the levels of CAB and/or RPV due to enzyme induction and therefore must not be administered concurrently:

•      Carbamazepine

•      Oxcarbazepine

•      Phenobarbital

•      Phenytoin

•      Rifabutin

•      Rifampicin / Rifampin

•      Rifapentine

•      St. John’s wort (Hypericum perforatum)

•      Systemic dexamethasone (>a single dose)

In addition to above, for participants receiving the oral formulation of RPV, the following medications could significantly decrease the levels of RPV due to gastric acid reduction and therefore must not be administered concurrently: proton pump inhibitors like esomeprazole, lansoprazole, omeprazole, pantoprazole and rabeprazole. Should a participant be unable to stop using these medications or switch to an acceptable alternative during RPV treatment, they should not be included in the study.

In addition, for participants receiving CAB LA and RPV LA, use of anticoagulation agents for greater than 14 days is prohibited, with the exception of the use of anticoagulation for deep vein thrombosis prophylaxis (e.g., postoperative deep vein thrombosis [DVT] prophylaxis) or the use of low dose acetylsalicylic acid (≤325 mg). Systemic anticoagulation (including prophylaxis doses) on the day of an IM injection should be avoided.

Note: Any prohibited medications that decrease cabotegravir or rilpivirine concentrations should be discontinued for a minimum of 4 weeks or a minimum of three half-lives (whichever is longer) prior to the first dose and any other prohibited medications should be discontinued for a minimum of 2 weeks or a minimum of three half-lives (whichever is longer) prior to the first dose.

### Study procedures

Study participants will undergo several clinical and laboratory assessments, complete various questionnaires and participate in qualitative interviews, as detailed in the schedule of activities (SoA) ( Table 2).

**Table 2.** IMPALA Trial Schedule of Activities.

Phase	Screening period ^a^		Optional OLI ^b^		Initiation (Months)	Maintenance Phase (Months)															
Period	SCR 1	SCR 2 ^a^	Baseline	1	2	3	4	6	8	9	10	12	14	15	16	18	20	21	22	24 ^c^	Unscheduled Visit ^t^
**Visit Window (days)**			**±8**	**±7**																	
Study visits – vary by arm, activities listed below only apply if the participant has a visit scheduled in line with their randomization arm																					
CAB LA + RPV LA			X	X	X		X	X	X		X	X	X		X	X	X		X	X	X
cART	X	(X) ^u^	X			X		X		X		X		X		X		X		X	
Clinical assessments and procedures																					
Informed consent / assessment of understanding	X																				
Administration of oral CAB and RPV			X ^b^																		
Administration of IM CAB/ RPV ^d^				X	X		X	X	X		X	X	X		X	X	X		X	X	X
Chest Xray, ECG	X																				
Assessment of eligibility	X	(X) ^u^																			
Medical history and demographics	X																				
Adherence assessment ^e^	X	(X) ^u^	X	X		X		X		X		X		X		X		X		X	X
Con-meds	X	X	X	X	X	X	X	X	X	X	X	X	X	X	X	X	X	X	X	X	X
Vital Signs	X	X	X	X	X	X	X	X				X				X				X	X
Physical examination	X		X					X				X				X				X	
ISR assessment				X	X		X	X	X		X	X	X		X	X	X		X	X	X
AE assessment ^f^	X	X	X	X	X	X	X	X	X	X	X	X	X	X	X	X	X	X	X	X	X
Weight and appetites	X		X					X				X ^s^				X				X ^s^	
Height			X																		
EuroQoL-SD-SL			X					X				X								X	
MOS-HIV			X					X				X								X	
HIVTSQs			X					X													
HIVTSQc												X								X	
Treatment preference												X								X	
Medical resource utilization and patient-related outcomes																					
MRU			X									X ^g^				X					
In-depth interviews ^h^			X					X				X									
OLI questionnaire (LA arm only)			X																		
Blood and urine tests																					
HIV-1 RNA ^i, j^	X	(X) ^iu^	X		X ^k^	X ^l^		X	X ^k^	X ^l^		X				X				X	
CD4+ count	X		X					X				X				X				X	
Hepatitis B serology (HBcAb, HBsAg, HBsAb)	X											X ^r^								X ^r^	
Hepatitis C serology (HCV Ab)	X																				
CBC, Biochemistry ^m^	X		X		X	X	X ^q^	X				X				X				X	
HbA1c, fasting lipids			X									X								X	
Urine HCG ^n^	X	(X) ^u^	X	X	X	X	X	X	X	X	X	X	X	X	X	X	X	X	X	X	X
Urine dipstick (protein, glucose)			X					X				X				X				X	
Store PBMC or whole blood ^o^			X																		
Store plasma ^p^	X		X		X ^k^	X ^l^		X	X ^k^	X ^l^		X				X				X	

**Abbreviations:** AE = adverse event; ART= antiretroviral therapy; CAB= cabotegravir; CBC = complete blood count; Con-meds = concomitant medications; ECG= electrocardiogram; FGDs = focus group discussions; HbA1c = hemoglobin A1c; HBcAb = hepatitis B core antibody; HBsAg = hepatitis B surface antigen; HBsAb = hepatitis B surface antibody; HCG= human chorionic gonadotrophin; HCVcAb = hepatitis C core antibody; HIVTSQc= HIV Treatment Satisfaction Questionnaire- change version; HIVTSQs= HIV Treatment Satisfaction Questionnaire- status version; IM= intramuscular; ISR= injection site reactions; LA=long acting; OLI=oral lead-in; PBMC= peripheral blood mononuclear cells; RPV=rilpivirine; SCR = screening. **Footnotes:** a. Screening visit 2 is only required if HIV-1 VL ≥200 at screening visit 1 or duration of virologic suppression (HIV-1 VL <200 c/mL) has not reached 3 months. Once a participant is confirmed as virologically suppressed (<200 c/mL), and duration is ≥3 months then randomization should occur within 2 weeks. The screening period must be completed within 4 months. a. One month of oral CAB and RPV (OLI) or continue daily oral ART (DTI) prior to first injections of CAB LA + RPV LA at month 1. b. Perform Month 24 assessments in all participants who withdraw from study intervention prematurely (if participant is willing), and scheduled assessments off the study intervention should be continued up to the Month 24 visit as specified in Section 7.1, Discontinuation of Study Intervention. c. For participants with BMI ≥30 a longer needle should be used as per product administration instructions. d. Adherence assessment and tablet count only in participants receiving daily oral ART, or oral lead-in/bridging treatment in CAB LA + RPV LA group. e. Clinical AEs will be captured from the time of screening. Laboratory abnormalities will not be recorded/reported as AEs till after the baseline visit. f. Detailed healthcare resource data survey to be performed at Month 12 visit, at selected sites only g. From a purposively selected sample at selected sites. h. HIV-1 VL can be repeated on one additional occasion after screening visit 1 if it is still ≥200 c/mL. i. Additional HIV-1 VL testing is performed after 4 (3–6) weeks in participants with VL ≥200 c/mL in CAB LA + RPV LA group; additional plasma sample stored (for batched, blinded viral load testing) after 4 (3–6) weeks in participants with VL ≥200 c/mL in the cART arm; and additional viral load after 12 (10–14) weeks in participants with viral load ≥1000 c/mL in the cART arm; reflex resistance testing is performed on stored plasma sample in participants with VL ≥200 c/mL in the CAB LA + RPV LA group, or ≥1000 c/mL in the cART group. (See also Section 8.1.1.2 for an alternative threshold for testing and definition of virologic failure in the cART group) Pregnant participants in the CAB LA + RPV LA arm who continue the study should have viral load monitoring frequency increased to every 2 months aligned with CAB LA + RPV LA Q2M injection visits during pregnancy and for 6 months following delivery or more frequently as per treating physician preference. Repeat HIV-1 VL testing should be performed after 2–3 weeks in pregnant participants with VL ≥50 c/mL. j. CAB LA + RPV LA arm only k. cART arm only l. Complete blood count: hemoglobin, total white cell count and differential, neutrophil count, platelets. Biochemistry: creatinine, sodium, potassium, alanine transaminase, alkaline phosphatase, total bilirubin, aspartate aminotransferase (at screening only). For individuals switched from NNRTI to dolutegravir during the screening period national guidelines should be followed regarding glycemic monitoring before and after switch. m. Only required for females of reproductive potential n. PBMC stored for baseline archived genotypic drug resistance testing. o. One large size (8–10 mL) EDTA sample stored at the following time-points: at screening, baseline visit, all visits where HIV VL is performed, and each visit where the blood is taken for confirmation of viral load rebound (for additional resistance testing, pharmacokinetic analysis, immune or biomarker tests; both arms); at 1, 3, 6, and 12 months following cessation of CAB LA + RPV LA in participants who develop TB (for drug levels; CAB LA + RPV LA group only); at 1 ^st^, 2 ^nd^, and 3 ^rd^ trimester and postpartum in pregnant women (for drug levels; in those continuing CAB LA + RPV LA only); in individuals consenting to peak drug level measurement, at 1 week (±1 day) post injection at Month 4, Month 6, Month 10, Month 16, Month 22, where feasible. p. Liver function tests (total bilirubin, alanine transaminase, alkaline phosphatase) should be repeated in those in the CAB LA + RPV LA arm who have a positive HBcAb during screening. q. Hepatitis B surface antigen (HBsAg) - only in those in the intervention arm who had positive hepatitis B core antibody (HBcAb) at screening. r. Appetite will be evaluated only at Month 12 and M-nth 24. s. An unscheduled visit to re-initiate CAB LA + RPV LA may occur between two 2-month interval visits following discontinuation due to pregnancy or TB. All the assessments at this visit are similar to those performed at the Month 1 initiation visit. A viral load measurement will need to be taken at the prior visit one month earlier and reviewed at this re-initiation visit to ensure viral suppression at re-initiation. t. “(X)” marks assessment/activity that has an impact on the inclusion/exclusion criteria of the participant.

### Laboratory procedures

Blood samples are collected for the following tests per the SoA ( Table 2): haematology (complete blood count), CD4+ T cell count, serum chemistry (sodium, potassium, creatinine, liver function tests [LFTs] including alanine transaminase (ALT), aspartate aminotransferase (AST), total bilirubin, alkaline phosphatase (ALP)), a metabolic profile (haemoglobin A1c (HbA1c), fasting cholesterol, fasting triglycerides) and random urine sample for urinalysis. For participants in the CAB LA + RPV LA arm with a positive screening hepatitis B core antibody (HBcAb), repeat LFTs are required at the Month 4 visit. In the control arm, individuals who are switched from NNRTI to DTG during the screening period undergo additional glucose monitoring if required by national guidelines.

The laboratory results are reviewed by the investigator who documents the review and records any clinically relevant changes occurring during the study in the Adverse event (AE) log. Urine pregnancy tests are performed at all study visits for female participants of reproductive potential.

For efficacy assessments, participants in both groups have routine HIV-1 VL monitoring at baseline and Months 6, 12, 18, and 24. Participants in the CAB LA + RPV LA Q2M arm also have HIV-1 VL monitoring at Months 2 and 8, and those in the oral ART control arm have additional HIV-1 VL monitoring at Months 3 and 9.

### Clinical assessment

Participants have vital signs including respiratory rate, heart rate and blood pressure measured at each visit during the screening period and the first six months of the study. These measurements are then taken again at Months 12, 18 and 24. Height is measured at the baseline visit to enable body mass index (BMI) calculations, and weight is measured at screening, baseline and Months 6, 12, 18 and 24.

At every visit, participants are assessed for any interim AEs. All clinical AEs are recorded from the first screening visit onwards, while asymptomatic laboratory AEs are reported from after baseline onwards. Post-injection reactions are considered AEs of special interest and will be recorded as such.

### Qualitative methods sub-study

As shown in the SoA, participants complete questionnaires on quality of life ^18, 19^ and treatment satisfaction ^20^ at baseline and at Months 6, 12 and 24. Additionally, participants in the CAB LA + RPV LA arm answer a single question regarding treatment preference at Months 12 and 24. All participants are asked to complete a brief questionnaire about medical resource utilisation at months 12 and 18 to support health economic analysis. A brief questionnaire regarding the choice of OLI versus DTI will be administered at baseline to all participants randomised to the CAB LA + RPV LA arm.

In-depth interviews are conducted with a subset of 10–20 individuals from both Uganda and South Africa, at baseline, Month 6, and Month 12. Participants provide separate consent to participant in this qualitative sub-study. Additionally, up to 15 individuals who experience virological failure in the CAB LA + RPV LA arm will be approached to participate in a single in-depth interview, using a separate participant information sheet and consent form.

### Pregnancy

All initial reports of pregnancy in female participants or partners of male participants must be reported to the sponsor by the study-site personnel within 24 hours of their knowledge of the event using the appropriate pregnancy notification form. Abnormal pregnancy outcomes (e.g., spontaneous abortion, foetal death, stillbirth, congenital anomalies, ectopic pregnancy) are considered Serious Adverse Events (SAEs) and must be reported using a SAE reporting form. Any participant from the CAB LA + RPV LA arm who becomes pregnant will be permitted to continue the study product after providing separate informed consent. Plasma samples for pharmacokinetic (PK) analyses will be taken from pregnant participants, for evaluation of CAB and RPV concentrations. The frequency of HIV-1 VL monitoring will increase to 2-monthly for the duration of the pregnancy, and all infants’ HIV-1 test results will be followed up.

### Measurement of CAB LA and RPV LA concentrations

Drug concentration measurements within the study may be useful in understanding how drug exposures vary according to demographic or anthropometric factors (such as sex, BMI or pregnancy), and for determining how drug concentrations impact treatment outcomes. Samples for measuring of drug levels are collected in various scenarios as outlined in the SoA, including during pregnancy, tuberculosis treatment, treatment overdose, inadvertent intravenous administration and virological failure. Peak (1 week post injection) and trough drug levels (collected pre-injection) will also be measured from stored plasma samples in a subgroup of participants.

### Sample size calculation

Assuming a true 78% virologic response rate in each group, a non-inferiority margin of -10%, and a 2-sided 5% significance level (one-sided 2.5% significance level), this study requires 270 participants per treatment group to provide 80% power to test the hypothesis of noninferiority of CAB LA + RPV LA Q2M vs. daily oral ART. Should non-inferiority be demonstrated, this sample size also provides 80% power to declare superiority of CAB LA + RPV LA Q2M compared with daily oral ART if the difference in virologic response between the treatment arms is 10% (83% CAB LA + RPV LA Q2M vs. 73% daily oral ART).

In addition, this sample size provides at least 80% power for the key secondary non-inferiority hypothesis for virologic failure, with a 2-sided 5% significance level, non-inferiority margin of 4% and assumed virologic failures rates of 4% in CAB LA + RPV LA and 6% in daily oral ART.

### Objectives and endpoints

The objectives and endpoints of the Study are shown in Table 3.

**Table 3.** Study Objectives and Endpoints.

Objectives	Endpoints
Primary:	
To demonstrate the non-inferior efficacy of switching to every 2 months (Q2M) intramuscular (IM) injection of cabotegravir (CAB) long-acting (LA) plus rilpivirine (RPV) LA compared with continuation of daily oral ART over 12 months in people living with HIV-1 (PLHIV1) with a history of sub-optimal ART adherence or engagement in care	• Proportion with plasma HIV-1 viral load (VL) <50 c/mL at 12 months (by Food and Drug Administration [FDA] snapshot algorithm)
Secondary:	
To demonstrate the antiviral activity and the impact on retention in HIV care of switching to Q2M CAB LA + RPV LA compared with continuation of daily oral ART over 12 and 24 months in PLHIV1 with a history of sub-optimal ART adherence or engagement in care	• Proportion with confirmed virologic failure (CVF) [plasma HIV-1 VL ≥200 c/mL on 2 consecutive occasions] at 12 and 24 months • Proportion with LTFU [>4 weeks elapsed since their last missed appointment] at 12 and 24 months • Proportion with plasma HIV-1 VL <200 c/mL at 12 months by FDA snapshot algorithm • Proportion with virologic response [plasma HIV-1 VL <50 c/mL] at 24 months by FDA snapshot algorithm • Proportion with virologic non-response [plasma HIV-1 VL ≥50 c/mL] at 12 and 24 months by FDA snapshot algorithm
To demonstrate the immunological activity of switching to Q2M CAB LA + RPV LA compared with continuation of daily oral ART over 12 and 24 months in PLHIV1 with a history of sub-optimal ART adherence or engagement in care	• Change in CD4+ T cell count from baseline (12 and 24 months) • Incidence of HIV disease progression (HIV/AIDS-related hospitalizations, illness, or deaths) (through 24 months)
To evaluate the safety and tolerability of switching to Q2M CAB LA + RPV LA compared to continuation of daily oral ART	• Incidence of adverse events through 12 and 24 months • Incidence of adverse events, Grade 3 and 4 (through 12 and 24 months) excluding injection site reactions (ISRs) • Frequency of ISRs of any grade
To assess genotypic viral resistance in participants experiencing protocol-defined CVF (confirmed plasma HIV-1 RNA ≥200 c/mL on 2 consecutive tests) and its impact on future treatment options including proportion who resuppress on dolutegravir	• Frequency of emergence of new integrase strand transfer inhibitor (INSTI) and non-nucleoside reverse transcriptase inhibitor (NNRTI) drug resistance mutations in those who develop virologic failure (through 12 and 24 months)
Exploratory	
To evaluate the effect of Q2M CAB LA + RPV LA on health-related quality of life	• Change from baseline in EQ-5D-5L at Month 12 and Month 24 • Change from baseline in MOS-HIV at Month 12 and Month 24 • Quality of life outcomes of in-depth interviews
To assess treatment satisfaction	• For participants in the CAB LA + RPV LA group, change from baseline in HIVTSQ scores at Month 6, Month 12 and Month 24
To assess preference for Q2M CAB LA + RPV LA compared to daily oral ART	• For participants randomized to the CAB LA + RPV LA group, preference for CAB LA + RPV LA compared to daily oral ART regimen at Month 12 and Month 24 using a single dichotomous preference question
To measure participants' medical resource utilization under Q2M CAB LA + RPV LA compared to daily oral ART	• Medical resource utilization over 24 months • Clinic/hospital attendances • Rates of opportunistic infections and other illnesses
To assess programmatic acceptability of Q2M CAB LA + RPV LA	• Thematic analysis of data from interviews participants, healthcare workers and key stakeholders
To evaluate adherence to treatment	• Self-reported adherence and pill count for daily oral ART and delayed/missed injections for CAB LA + RPV LA
To evaluate drug levels	• Plasma concentrations of CAB LA and RPV LA

## Statistical Analysis Plan

### Primary Statistical Hypothesis

CAB LA + RPV LA is non-inferior (non-inferiority margin, -10%) in the proportion of participants with plasma HIV-1 RNA <50 c/mL as per FDA snapshot algorithm at Month 12, compared to a daily triple drug oral regimen consisting of 2NRTI + DTG regimen (ART).

Analysis will be undertaken to evaluate primary and secondary objectives of the protocol after all participants have completed their Month 12 visit, and undertaken again once all participants have completed their Month 24 visit. The primary analysis will be conducted using the Month 12 data. No adjustment for multiplicity resulting from repeated evaluation of the primary endpoint will be made, as the Month 24 analyses will be secondary.

For the primary efficacy endpoint analysis, each participant’s virologic response (HIV 1 RNA <50 c/mL) will be calculated according to the FDA’s snapshot algorithm. The primary analysis will be based on the intention to treat (ITT) analysis set. For the primary comparison, the adjusted estimate of the difference in the proportion of participants with virologic response between the 2 groups will be presented along with confidence intervals (CIs), based on an analysis stratified by study site using the Cochran Mantel-Haenszel Method (CMH). Treatment with CAB LA + RPV LA will be considered non-inferior to daily oral ART if the lower limit of the CMH-based 95% CI of the difference in virologic response between the treatment groups is >-10%. Should treatment with CAB LA+ RPV LA demonstrate non-inferiority to daily oral ART, an additional hypothesis testing on the superiority of CAB LA + RPV LA will be conducted. CAB LA+ RPV LA will be considered superior to daily oral ART if the lower limit of the CMH-based 95% CI of the difference in virologic response between the treatment groups is >0%. Since an analysis on the ITT analysis set may not be conservative in a non-inferiority setting, an analysis based on the PP analysis set will also be performed to investigate the impact of excluding participants with major protocol violations and evaluate the robustness of the primary analysis results.

The key secondary endpoint of CVF at Month 12 will be analysed to test the non-inferiority hypothesis. The adjusted estimate of the difference in the proportion of participants with CVF between the 2 groups will be presented along with CIs, based on an analysis stratified by study site using the CMH method. Treatment with CAB LA + RPV LA will be considered non-inferior to daily oral ART if the upper limit of the CMH-based 95% CI of the difference in CVF between the treatment groups is <4%. For virologic endpoints at Month 24, analyses will be conducted similarly as for Month 12.

The independent data safety monitoring committee (IDMC) will evaluate the interim efficacy, tolerability, and safety of CAB LA + RPV LA for this study. The IDMC will review safety data on a rolling 6-monthly interval. An interim futility analysis will take place once 50% of trial participants have data available on the primary virologic response outcome at Month 12. A Haybittle-Peto stopping rule will be pre-specified such that the trial will be stopped only if futility is demonstrated beyond reasonable doubt (i.e., P<0.001), with no adjustment made to the overall study level of significance for the final analysis (Alpha=0.05). The statistical analysis plan (SAP) will describe the planned interim analyses in greater detail, and additional details regarding the IDMC will be provided separately in the IDMC charter.

### Safety analyses

The verbatim terms used in the eCRF by investigators to identify AEs will be coded using the Medical Dictionary for Regulatory Activities (MedDRA). Any AE occurring at or after the initial administration of study intervention through the day of last dose plus 30 days is considered treatment emergent. All reported treatment-emergent AEs will be included in the analysis. For each AE, the percentage of participants who experience at least 1 occurrence of the given event will be summarized by the intervention group. Summaries, listings, datasets, or participant narratives may be provided, as appropriate, for participants who die, discontinue intervention due to an AE, or experience severe AEs or SAEs.

## Ethical considerations

Potential participants are fully informed of the potential benefits, risks and requirements of the study. During the study, participants are given any new information that may affect their decision to continue participation. Participants will be informed that their consent to participate in the study is voluntary and may be withdrawn at any time with no reason given and without penalty or loss of benefits to which they are otherwise be entitled. Only those who understand the risks and benefits and pass the assessment of understanding questions are eligible to provide voluntary consent for enrolment. The current ART regimen administered in this protocol consists of 2 NRTIs plus DTG as the third agent. These regimens are established treatments that are recommended first-line options and, have been in clinical use for several years. Detailed information on their well-established benefit/risk profiles, together with risk mitigation measures, are described in their respective local product labels.

The trial Sponsor is responsible for ensuring the Trial is appropriately indemnified and has the necessary Public Liability and Clinical Trial insurance policies in place.

All study teams are committed to adhering to protocol-specified assessments for all participants. Modifications to protocol-required assessments may be permitted after consultation between the participant and investigator, and with the agreement of the sponsor and a protocol amendment being approved. Missed assessments/visits are captured in the clinical trial management system as protocol deviations. All protocol deviations are reported to respective site research ethics committees as per country specific guidelines.

### Study monitoring

Study monitoring is being carried out to ensure the protection of participants' rights and well-being, confirm that the data collected is accurate, complete, and verifiable from source documents, and ensure that the trial is conducted according to the trial protocol, ICH-GCP guidelines, and relevant regulatory requirements. The trial monitoring plan, which outlines the monitoring activities before, during, and after the trial, was finalized and approved by the sponsor before the trial started. Monitoring activities include a site initiation visit for each site prior to the start of screening, regular on-site monitoring visits during the trial, and a close-out visit. In addition, remote monitoring of the trial database is conducted on a daily basis by the data manager and trial safety lead. The study has an independent data monitoring committee (IDMC) who reviews the accumulating safety data in line with the approved IDMC charter.

The Trial Safety Group monitors safety on a regular basis through the creation and review of regular trial safety reports and clinical review of adverse event data.

### Participant confidentiality

All reports and communications relating to the study will identify participants by the participant identification number and will use age at initial informed consent. In cases where the participant is not randomized into the study the date seen and age at initial informed consent will be used.

## Plans for dissemination

The results of the study will be reported in a Clinical Study Report generated by the Sponsor which will include data from all study sites. Participant identifiers will not be used in the publication of results. Any analyses performed after the Clinical Study Report is issued will be reported separately. It is the intent to present the results at conferences and published in peer-reviewed journals. A detailed publication plan is being developed and will be reviewed by the Trial Steering Committee and Investigators. The MRC/UVRI and LSHTM Uganda Research Unit Community Advisory Board (CAB) has been informed about the study, and ongoing engagement with the CAB and other collaborating institutions will continue throughout the study.

## Ethical approval and consent statement

This study is being carried out across 7 sites in Uganda, Kenya, and South Africa.

In Uganda the study was granted approval by the Uganda National Council for Science and Technology (HS2475ES, 02 ^nd^ November 2022) the Uganda Virus Research Institute Research Ethics Committee (GC/127935, 27 ^th^ October 2022) and the National Drug Authority (0224/2022, 7 ^th^ November 2022). Approval was also granted by the London School of Hygiene & Tropical Medicine Research Ethics Committee (28325, 21 ^st^ November 2022).

In Kenya ethical approval was granted by the Kenyatta National Hospital University of Nairobi Ethics Review Committee (KNH/ERC/A/25, 16 ^th^ January 2023) and the Jaramogi Oginga Odinga Teaching and Referral Hospital Institutional Scientific and Ethical Review Committee (ISERC/JOOTRH/667/22, 18 ^th^ January 2023). Approval was also granted by the Pharmacy and Poisons Board (ECCT/23/01/05, 14 ^th^ March 2023).

In South Africa the study received approval from the University of KwaZulu Natal Biomedical Research Ethics Committee (BREC/00005310/2023, 2 ^nd^ June 2023), the University of Cape Town Research Ethics Committee (041/2023, 16 ^th^ May 2023) and the South African Health Products Regulatory Authority (20230201, 23 ^rd^ Mar 2023).

All participants were required to sign a written informed consent form outlining the benefits, risks, procedures and reimbursement relating to the study. Illiterate participants were allowed to use a thumbprint in the presence of an impartial witness. Separate informed consent forms were used for sample storage, pregnancy and the peak pharmacokinetic sub-study where appropriate. Screening of potential participants commenced on 8 ^th^ December 2022. The first participant was enrolled on 6 ^th^ January 2023 and the last participant was enrolled on 6 ^th^ May 2024. Participant follow-up is ongoing.

## Data Availability

Data may be available to researchers following the completion of the study and the main publication in accordance with the approved Data Management Plan. Researchers wishing to access the data should contact the TMG in the first instance via the trial administration email at impala@lists.lshtm.ac.uk. All data exchange must comply with information Governance and Data Protection Policies applicable in all relevant countries. No data are associated with this article currently. Zenodo: SPIRIT checklist for: Efficacy and safety of long acting injectable cabotegravir and rilpivirine in improving HIV-1 control in Sub Saharan Africa: Protocol for a phase 3b open label randomized controlled trial (IMPALA). DOI https://doi.org/10.5281/zenodo.14799838 ^21^. This data is held under Creative Commons Attribution 4.0 International (CC-BY 4.0).
