# Supplementary material for: Efficacy and Safety of Long-acting Injectable Cabotegravir and Rilpivirine in Improving HIV-1 Control in sub-Saharan Africa: Protocol for a Phase 3b Open-Label Randomized Controlled Trial (IMPALA)
Source: Wellcome Open Res. 2025 Mar 28;10:166. [Version 1] doi: 10.12688/wellcomeopenres.23363.1 (PMC12485332; doi:10.12688/wellcomeopenres.23363.1)
Supplement: Peer review history

# Reviewer report: Reviewer response for version 1

**DOI:** 10.21956/wellcomeopenres.25753.r122039

**Published:** 2025-05-02

**Authors:** Stefano Rusconi

**Affiliations:** 1 Universita' degli Studi di Milano, Milan, Italy

The study protocol aims at responding to a pivotal question in clinical research: can long-acting agents be useful for treating PWH with a history of sub-optimal adherence to daily oral ART. The protocol is directed to PWH living in SSA and the follow-up period will be 24 months.

 I agree with the exclusion of DTG-based dual therapy, after considering the group of PWH with a sub-optimal adherence. in the same area, I suggest to control for the (optional) oral lead-in therapy. Although, it was not a criterion in phase III studies, I think it will be better to assess this variable in this group of PWH with a sub-optimal adherence.

Is the study design appropriate for the research question?

Yes

Is the rationale for, and objectives of, the study clearly described?

Yes

Are sufficient details of the methods provided to allow replication by others?

Yes

Are the datasets clearly presented in a useable and accessible format?

Yes

Reviewer Expertise:

Infectious diseases; new compounds in vivo and in vitro.

I confirm that I have read this submission and believe that I have an appropriate level of expertise to confirm that it is of an acceptable scientific standard.

# Reviewer report: Reviewer response for version 1

**DOI:** 10.21956/wellcomeopenres.25753.r122040

**Published:** 2025-04-16

**Authors:** Eisuke Adachi

**Affiliations:** 1 The University of Tokyo, IMSUT Hospital, Tokyo, Japan

This is a highly significant randomized controlled trial investigating CAB+RPV in individuals with sub-optimal adherence, particularly in a resource-limited setting and among PWH with viremia.

 Although participants with HIV-RNA <200 copies/mL were included, the study design does not explicitly exclude individuals who were already virologically suppressed at the time of treatment initiation. Since the screening criteria are key to defining the study population, I recommend clarifying this in the manuscript.

 1. Line 1 of the Protocol Study Design section:

 Please revise the sentence to:

*“IMPALA is a randomized controlled open-label interventional study among virologically suppressed (HIV-1 viral load [VL] <200 copies/mL) adults (≥18 years) who have a history of protocol-defined sub-optimal adherence to daily oral ART and/or engagement in HIV care (Table 1).”*

 2. Additionally, please include a rationale for excluding individuals receiving DTG/3TC regimens. Was this due to the inclusion of participants with viremia?

Is the study design appropriate for the research question?

Yes

Is the rationale for, and objectives of, the study clearly described?

Yes

Are sufficient details of the methods provided to allow replication by others?

Yes

Are the datasets clearly presented in a useable and accessible format?

Yes

Reviewer Expertise:

HIV, clinical trials, STD

I confirm that I have read this submission and believe that I have an appropriate level of expertise to confirm that it is of an acceptable scientific standard.
